# Immunohistochemical distribution of 10 GABA_A_ receptor subunits in the forebrain of the rhesus monkey *Macaca mulatta*

**DOI:** 10.1002/cne.24910

**Published:** 2020-04-03

**Authors:** Günther Sperk, Elke Kirchmair, Jaco Bakker, Werner Sieghart, Meinrad Drexel, Ivanela Kondova

**Affiliations:** ^1^ Department of Pharmacology Medical University Innsbruck Innsbruck Austria; ^2^ Division of Veterinary Care, Animal Science Department Biomedical Primate Research Centre Rijswijk The Netherlands; ^3^ Department of Molecular Neurosciences, Center for Brain Research Medical University Vienna Vienna Austria; ^4^ Division of Pathology and Microbiology, Animal Science Department Biomedical Primate Research Centre Rijswijk The Netherlands

**Keywords:** antibody α1 (BD24), RRID:AB_2108828, ab α2, RRID:AB_2827793, ab α3, RRID:AB_2827797, ab α4, RRID:AB_2827798, ab α5, RRID:AB_2827799, ab β1, RRID:AB_2827800, ab β2, RRID:AB_2827801, ab β3, RRID:AB_2827802, ab γ2, RRID:AB_2827803, ab δ, RRID:AB_2827804, ab GAD67, RRID:AB_2278725, ab NeuN, RRID:AB_2278725, amygdala, basal ganglia, benzodiazepine, GABA_A_ receptor subunits, goat biotinylated anti‐rabbit ab, RRID:AB_2336810, horse anti‐mouse ab, RRID:AB_2336811, immunohistochemistry, monkey, primate, thalamus

## Abstract

GABA_A_ receptors are composed of five subunits arranged around a central chloride channel. Their subunits originate from different genes or gene families. The majority of GABA_A_ receptors in the mammalian brain consist of two α‐, two β‐ and one γ‐ or δ‐subunit. This subunit organization crucially determines the physiological and pharmacological properties of the GABA_A_ receptors. Using immunohistochemistry, we investigated the distribution of 10 GABA_A_ receptor subunits (α1, α2, α3, α4, α5, β1, β2, β3, γ2, and δ) in the fore brain of three female rhesus monkeys (*Macaca mulatta*). Within the cerebral cortex, subunits α1, α5, β2, β3, and γ2 were found in all layers, α2, α3, and β1 were more concentrated in the inner and outer layers. The caudate/putamen was rich in α1, α2, α5, all three β‐subunits, γ2, and δ. Subunits α3 and α5 were more concentrated in the caudate than in the putamen. In contrast, α1, α2, β1, β2, γ2, and δ were highest in the pallidum. Most dorsal thalamic nuclei contained subunits α1, α2, α4, β2, β3, and γ2, whereas α1, α3, β1, and γ2 were most abundant in the reticular nucleus. Within the amygdala, subunits α1, α2, α5, β1, β3, γ2, and δ were concentrated in the cortical nucleus, whereas in the lateral and basolateral amygdala α1, α2, α5, β1, β3, and δ, and in the central amygdala α1, α2, β3, and γ2 were most abundant. Interestingly, subunit α3‐IR outlined the intercalated nuclei of the amygdala. In the hippocampus, subunits α1, α2, α5, β2, β3, γ2, and δ were highly expressed in the dentate molecular layer, whereas α1, α2, α3, α5, β1, β2, β3, and γ2 were concentrated in sector CA1 and the subiculum. The distribution of GABA_A_ receptor subunits in the rhesus monkey was highly heterogeneous indicating a high number of differently assembled receptors. In most areas investigated, notably in the striatum/pallidum, amygdaloid nuclei and in the hippocampus it was more diverse than in the rat and mouse indicating a more heterogeneous and less defined receptor assembly in the monkey than in rodent brain.

## Graphical abstract

GABA_A_ receptors are composed of five subunits arranged around a central chloride channel. The different subunits originate from different genes or gene families. In the mammalian brain, they generally consist of two α‐, two β‐ and one γ‐ or δ‐subunit. The subunit organization crucially determines the physiological and pharmacological properties of the GABA_A_ receptors. Here we investigated the distribution of 10 different GABA_A_ receptor subunits in the forebrain of rhesus monkeys. We observed highly heterogeneous distribution patterns of the individual subunits indicating a high number of heterogeneously assembled GABA_A_ receptors. Their distribution was, however, less distinct in the monkey than in rodents.

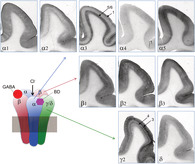

## 1 INTRODUCTION

GABA_A_ receptors are composed of five subunits arranged around a central chloride channel. Their subunits derive from different genes or gene families (α1–α6, β1–β3, γ1–γ3, δ, ε, θ). In the mammalian brain, the majority of GABA_A_ receptors consist of two α‐, two β‐, and one γ‐ or δ‐subunit. This subunit composition crucially determines the physiological and pharmacological properties of the individual GABA_A_ receptor subtypes (Olsen & Sieghart, 2009). Benzodiazepines and the hypnotic substance zolpidem are positive allosteric modulators of GABA_A_ receptors containing two α‐subunits (either α1, α2, α3, or α5), two β‐subunits (β1, β2, or β3) together with one γ‐subunit (not δ). They exert their action via the benzodiazepine‐binding site, located at the interface of an α‐ and the γ‐subunit. In contrast, the binding sites for GABA are located at the extracellular interface between an α‐ and a β‐subunit. The binding sites for neurosteroids still have not been finally identified. Neurosteroids may stimulate GABA_A_ receptors via binding sites located in the transmembrane α/β interface (Chen et al., 2019; Wu et al., 2019; Ziemba et al., 2018).

In rodents, the δ subunit specifically assembles with the α6‐subunit in the cerebellum and α4 and α5 in the thalamus, neostriatum, and the dentate gyrus (Farrant & Nusser, 2005; Glykys & Mody, 2007). These receptors are either extrasynaptically or perisynaptically located and mediate *tonic inhibition*, whereas α1‐, α2‐, and γ2‐containing receptors are positioned within the synapse and respond to release of GABA with *phasic inhibition* (Brickley & Mody, 2012; Glykys, Mann, & Mody, 2008; Glykys & Mody, 2007). Related to the expanded distance of diffusion of GABA, δ‐subunit containing receptors exert a higher affinity to GABA than receptors containing the γ2‐subunit. In rodent models of temporal lobe epilepsy, expression of the δ‐subunit is down regulated in the dentate gyrus, decreasing the responsiveness to tonic inhibition (Nishimura et al., 2005; Schwarzer et al., 1997; Tsunashima, Schwarzer, Kirchmair, Sieghart, & Sperk, 1997; Zhang, Wei, Mody, & Houser, 2007).

Furthermore, functioning of the GABA_A_ receptor/chloride channel is highly dependent on the interplay of two different chloride transporters, NKCC1 and KCC2 (Ben‐Ari, 2014). Depending on the chloride gradient, stimulation of GABA_A_ receptors can mediate hyperpolarization (chloride influx) or depolarization (chloride efflux) of the neuron. In the healthy mature brain, KCC2 is mostly more active and transports chloride outside, whereas, in the prenatally developing brain, NKCC1 predominates resulting in increased inward transport of chloride ions, which leads to depolarization of the neuron upon stimulation of the receptor.

The distribution of individual GABA_A_ receptor subunit mRNAs and proteins has been examined in detail by immunohistochemistry or in situ hybridization in rodents (Fritschy & Mohler, 1995; Hörtnagl et al., 2013; Laurie, Wisden, & Seeburg, 1992; Pirker, Schwarzer, Wieselthaler, Sieghart, & Sperk, 2000; Tsunashima et al., 1997; Wisden, Laurie, Monyer, & Seeburg, 1992). Studies on the distribution of individual GABA_A_ receptor subunits in the human brain and in nonhuman primates are less complete and mostly include only a limited number of subunits. Thus, Waldvogel et al. established the immunocytochemical distribution of subunits α1–α3, β2/β3, and γ2 in the striatum (Waldvogel & Faull, 2015), substantia nigra (Waldvogel et al., 2008), thalamus (Waldvogel, Munkle, van Roon‐Mom, Mohler, & Faull, 2017), in the spinal cord in humans (Waldvogel et al., 1990), and in the basal ganglia of the baboon (Waldvogel, Fritschy, Mohler, & Faull, 1998). Recently, Stefanits et al. (2018) performed a comprehensive study of seven GABA_A_ receptor subunits in the human amygdala and hippocampus (α1, α2, α3, α5, β2, combined β2/3, and γ2). GABA_A_ receptor subunits have also been investigated in the diseased human brain. Thus, changes in the expression of subunits α1, α2, α3, the β‐subunits, and γ2 were reported in the hippocampus (Loup, Wieser, Yonekawa, Aguzzi, & Fritschy, 2000; Pirker et al., 2003), amygdala and entorhinal cortex of patients with temporal lobe epilepsy (Stefanits et al., 2019) and Alzheimer's disease (Kwakowsky et al., 2018), and expression of the α3 subunit is increased in the cerebral cortex and white matter of epilepsy patients with cortical dysplasia (Loup, Picard, Yonekawa, Wieser, & Fritschy, 2009). Waldvogel and Faull (2015) demonstrated pronounced changes in the expression of subunits α1, α2, α3, β2/3, and γ2 in the basal ganglia of Huntington's disease patients depending on the cellular localization of these subunits and Stojanovic et al. (2016) investigated changes in the expression of GABA_A_ receptor subunits in rhombencephalic structures during normal human brain development.

To provide a basis for further neuropathological studies in human and nonhuman primate brains and for comparing the distribution of GABA_A_ receptor subunits in the rodent and primate brain we now performed a comprehensive immunohistochemical study on 10 different GABA_A_ receptor subunits (α1, α2, α3, α4, α5, β1, β2, β3, γ2, and δ) in the forebrain of the rhesus macaque.

## 2 METHODS

### 2.1 Animals and tissue fixation procedure

Three healthy, retired, adult, female, rhesus macaques (9,160, Ri6004, and 9,029) were selected for this study following the Veterinarian's recommendation. The monkeys were 16, 8, and 20 years old and raised and housed in natural harem groups at the Biomedical Primate Research Centre, Rijswijk, Netherlands. The procedures performed in this study were in accordance with the Dutch laws on animal experimentation, with the regulations for animal handling as described in the EU Directive 63/2010. Data are shown for the youngest, 8 years old monkey and were confirmed in sections of the two other monkeys.

The monkeys were deeply anesthetized with a mixture of ketamine (15 mg/kg) and medetomidine (20 μg/kg) applied i.m., followed by buprenorphine (20 μg/kg i.m). After opening of the thoracic cavity, the pericard was removed, and a cannula was inserted through the left ventricle into the aorta. The descending aorta was clamped just above the diaphragm. Using a syringe pump (Type S2; Medima), the brain was perfused with around 400 ml (50 ml/min) phosphate‐buffered saline pH 7.4 (PBS) including heparin 25.000 IU at room temperature until the out‐coming perfusate was clear. We then switched to 4% paraformaldehyde (PFA) in PBS kept in an ice bath and continued perfusion with about 1.5 L for 20 min. Consecutively, the brains were removed and postfixed in 200 ml ice‐cold 4% PFA for 90 min. The brains were then transferred to a beaker containing 200 ml 10% sucrose in PBS and were kept overnight at 4°C. They were then transferred to 20% sucrose/PBS and kept for 5 hr at 4°C. After changing in 20% sucrose/PBS, the brains were put into vessels with sucrose, sealed, and sent together with cooling elements to the Innsbruck lab.

**FIGURE 1:**
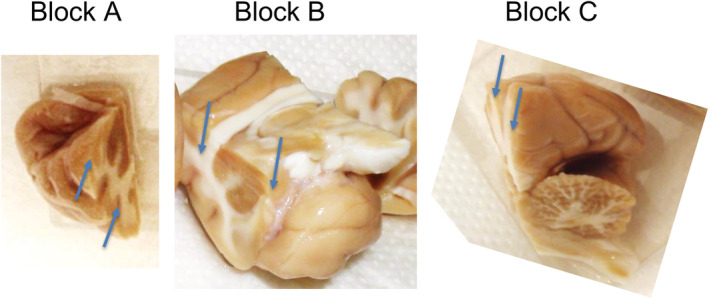
Dissection of the monkey brains. The paraformaldehyde‐fixed brains were first divided by a midsagittal cut into two hemispheres, and then each hemisphere was split into three parts by two coronal cuts at the intraaural Plane 0, (frontal to the cerebellum) and Plane 22 (frontal to the optic chiasm). Shown are the three blocks from one hemisphere. Block B was used for the experiments [Color figure can be viewed at wileyonlinelibrary.com]

In Innsbruck, we separated the perfused brains by midsagittal cuts into two hemispheres and then, each hemisphere was divided by two coronal cuts at the intraaural Plane 0, (frontal to the cerebellum) and Plane 22 (frontal to the optic chiasm), respectively, into three parts as shown in Figure 1. The brain pieces were snap‐frozen by immersing them into −70°C isopentane (Merck, Darmstadt, Germany) for 3 min. They were then transferred to an −80°C freezer and kept there for 48 hr to allow the isopentane to evaporate. Afterward, the frozen brain parts were sealed in tight plastic vessels and kept at −80°C until they were cut for immunohistochemistry.

### 2.2 Antibodies to GABA_A_ receptor subunits

The antibodies were originally raised against peptides corresponding in their sequences to the rat sequences of the GABA_A_ receptor subunits (see Table 1, and Sperk, Schwarzer, Tsunashima, Fuchs, & Sieghart, 1997). We had characterized them in our previous studies in the rat (Pirker et al., 2000; Pirker et al., 2003; Sperk et al., 1997). Monkey sequences of subunits α4, α5, and δ differed from the corresponding rat sequences (see shaded amino acids in the respective peptide sequences in Table 1). They were, however, identical with the respective human sequences. We, therefore, verified the specificity of the labeling by including synthetic peptides (DgPeptides Co., Hangzhou, China) for the corresponding human/macaque sequences of α5 and δ subunits at 100 nM in sections of the hippocampus. In the presence of the respective peptides, the labeling of subunits was entirely prevented (not shown).

**TABLE 1:** Antibodies and peptides. Peptide sequences derived from the respective rat subunit amino acid sequence are shown. The bold amino acids in α4, α5, and δ subunit sequences indicate differences to the human sequences. Below, the sequences of peptides used for characterization of α5 and δ antibodies are shown. The amino acid sequences correspond to the sequence of the macaque/human subunits, and the bold areas indicate differences to the rat. KP: The antibodies were raised against a peptide that was coupled to keyhole‐limpet hemocyanin; MBP: The peptide used for immunization was a fusion protein with maltose binding protein. The numbers of the amino acids include the amino acid sequences of the signal peptides.

Subunit	Ab conc. (μg/ml)	Gene bank no (human)	Amino acid No. monkey/human	RRID numbers	Sequence of peptides used for immunization (derived from the rat proteins)	Coupled to
α1	4.0	BD24		RRID:AB_2108828	Monoclonal Ab directed against purified human protein	
α2	1.5	P47869.2	350–385	RRID:AB_2827793	VNDKKKEKGSVMIQNNAYAVAVANYAPNLSKDPVLS	KP
α3	5.6	P34903.1	29–39	RRID:AB_2827797	QGESRRQEPGD	KP
α4	3.0	P48169.2	408–450	RRID:AB_2827798	NHSSK**TTAA**QESS**ET**TP**KAH**LASSPNPFSRANAAETISAAAR**G**	MBP
α5	4.0	P31644.1	368–418	RRID:AB_2827799	E**L**ILNKSTNAFTTGK**LT**HPPNIPKEQ**L**P**G**GT**G**N**AVG**T**A**S**IRA**SEEKTSES	KP
β1	4.5	P18505.2	375–400	RRID:AB_2827800	RNETSGSEVLTGVSDPKATMYSYDSA	MBP
β2‐2	3.4	NP000804	375–428	RRID:AB_2827801	NEMATSEAVMGLGDPRSTMLAYDASSIQYRKAGLPRHSFGRNALERHVAQKKSR	MBP
β3	1.6	P28472.1	370–429	RRID:AB_2827802	APMDVHNEMNEVAGSVGDTRNSAISFDNSGIQYRKQSMPKEGHGRYMGDRSIPHKKTHLRRRSS	MBP
γ2	1.4	P18507.2	358–405	RRID:AB_2827803	YFVSNRKPSKDKDKKKKNPAPTIDIRPRSATIQMNNATHLQERDEEYG	MBP
δ	2.3	O14764.2	17–60	RRID:AB_2827804	**QPHHGA**RAMNDIGDYVGSNLEISWLPNLDGL**ME**GYARNFRPGIG	MBP
Sequence of peptides used for characterization of antibodies (derived from the macaque/human proteins)						
α5			317–347		E**V**I**L**NKSTNAFTTGK**MS**HPPNIPKEQ**T**P**A**GT	
δ			25–59		MNDIGDYVGSNLEISWLPNLDGL**IA**GYARNFRPGI	

### 2.3 Immunohistochemistry

**FIGURE 2:**
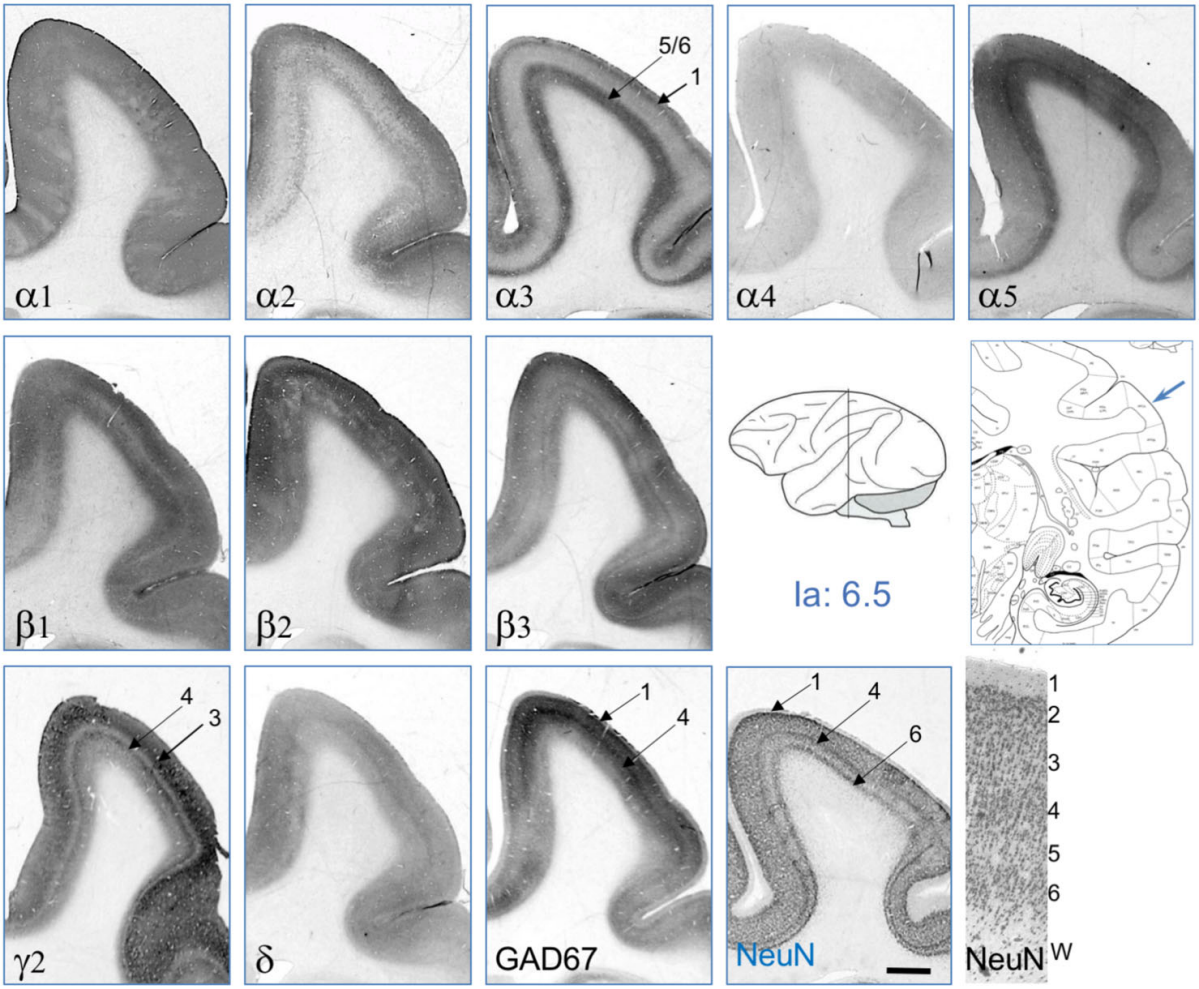
GABA_A_ receptor subunits in the cerebral cortex. Serial sections were taken at the intraaural level (Ia) 6.5, shown in the insert depicting a schematic monkey brain (adapted after Paxinos, Huang, Petrides, and Toga (2009). Shown are immunoreactivities for 10 GABA_A_ receptor subunits, GAD67, and NeuN. The figure contains also a graphical outline of the respective section to show the location of the cortical gyrus. For illustrating the cortical layers, NeuN‐IR is also shown at higher magnification (lower right panel). Scale bar = 3.0 mm [Color figure can be viewed at wileyonlinelibrary.com]

Only the “middle” brain pieces between Planes 0 (frontal to the cerebellum) and 22 (optic chiasm) were used (Block B in Figure 1). We virtually divided Block B (from one hemisphere) into 18 subfields, finally depicted in nine figures (Figures 2 and 4, 5, 6, 7, 8, 9, 10, 11). We obtained 30 μm thick serial coronal cryotome sections (MICROM HM 560; Histocom Medizintechnik Vertriebs GmbH, Wiener Neustadt, Austria) and collected 14 serial sections in 100 ml plastic vessels containing 0.1 M Tris‐buffered saline, pH 7.4 (TBS), 0.1% sodium azide and kept them sealed at 4–6°C. This resulted in 18 vessels containing subsequent coronal sections and representing one subfield of the monkey's brain each. Incubations were done with the 10 different receptor subunit antibodies, and the antibodies for glutamate decarboxylase 67 (GAD67) and neuron‐specific nuclear protein (NeuN). For incubations with each antibody, we took one section from each of the 18 vessels and incubated these sections concomitantly with the respective antibody. (For technical reasons, this was actually done in two incubations containing nine sections each, as described below). By this, we ended up with 18 matching serial sections for each antibody. Thus, at the end, it was possible to match each of these sections anatomically with 11 corresponding sections incubated with the other antibodies.

The primary antisera used for the GABA_A_ receptor subunits including their RRIDs are shown in Table 1. The following dilutions were used from the rabbit antibodies: 1:100 for α2, α4, α5, β1; 1:150 for α3, β2; 1:200 for γ2 and δ, and 1:300 for β3. For immunohistochemistry of the α1‐subunit, we used the commercially available monoclonal mouse antibody BD24 (1:250; Millipore Cat# MAB339, RRID:AB_2108828). For NeuN, we applied a monoclonal mouse antibody (1:10.000, Millipore Cat# MAB377, RRID:AB_2298772) obtained through Sigma‐Aldrich (Vienna, Austria) and for GAD67 a monoclonal mouse antibody (1:15.000, Millipore Cat# MAB5406, RRID:AB_2278725; IgG2α, clone1G10.2) obtained through Sigma‐Aldrich.

For incubation with each of the antibodies, nine sections (in steps covering the dorsal and ventral part of Block B, respectively) were separately transferred to eye cup‐like vessels containing 3.0 ml PBS. The sections were pretreated with target retrieval solution (pH 6.0; Dako, Vienna, Austria, 70°C, 20 min) and, after washing in 50 mM TBS pH 7.2 for 5 min, with 0.6% H_2_O_2_, 20% methanol/TBS for 20 min to reduce endogenous peroxidase activity. They were then incubated in 10% normal horse serum (GIBCO #26050–88, obtained through Fisher Scientific, Vienna, Austria) for GABA_A_ receptor α1‐subunit, GAD67, and NeuN or 10% normal goat serum (GIBCO #16210–072, obtained through Fisher Scientific) for the other GABA_A_ receptor subunits in TBS for 90 min and subsequently with the primary antiserum at 4°C for 48–72 hr. The sections were then processed by the VECTASTAIN ABC standard procedure using 1:200 dilutions of VECTASTAIN PK4002 horse biotinylated anti‐mouse antibody (Vector Laboratories Cat# PK‐4002, RRID:AB_2336811) for detecting the GAD67, NeuN, or GABA_A_ receptor α1‐subunit mouse antibodies, and VECTASTAIN PK4001 goat biotinylated anti‐rabbit antibody (Vector Laboratories Cat# PK‐4001, RRID:AB_2336810, obtained through Szabo‐Scandic, Vienna, Austria) for detecting the rabbit GABA_A_ receptor antibodies. Incubations with the biotinylated secondary antibodies and subsequent incubations with the ABC reagent (a mixture of avidin–biotin–horseradish peroxidase complex; 1:100) were done at room temperature for 60 min. The resulting complex was labeled by reacting the peroxidase with 0.05% 3,3′‐diaminobenzidine (Sigma, Munich, Germany) and 0.005% H_2_O_2_ (30%, Merck) in TBS for 4 min.

After each incubation step (except preincubation with 10% blocking serum), three 5 min washes with TBS were included. All buffers and antibody dilutions, except those for washing after target retrieval solution and peroxidase treatment, and the reaction with diaminobenzidine, contained 0.1% Triton X‐100. Normal horse or goat serum (10%) was included in all antibody‐containing buffers. Sections were mounted on glass slides, air‐dried, dehydrated, and cover‐slipped (coverslips: 60 × 45 mm^2^, VWR International, Vienna, Austria). Sections that had not been exposed to the primary antibody were included as controls and did not show any immunoreactive elements.

After each incubation, we collected the supernatant solution with the respective primary antibody, stored it at +4–6°C and reused it for three further incubations after complementing it each time by adding about 10% of the original antiserum (shown in Table 1). By this, we obtained identical labeling in the subsequent incubations.

### 2.4 Assessment of immunoreactivities

Semiquantitative assessment of the immunoreactivity, as shown in Table 3, was done by eye from images of the stained brain sections and by two independent investigators. Ratings +++, ++, and + refer to high, medium, and low immunoreactivity compared to the area with the highest immunoreactivity of the given subunit. These represent in general labeling of dendritic fields. Since the different antibodies exhibit different avidities, comparisons between different subunits cannot or only cautiously been drawn. The patterns of subunit distribution were equal in all three monkeys. Presumably, due to somewhat less stringent fixation, labeling was best in the 8 years old macaque. Data are therefore shown for this animal.

## 3 RESULTS

### 3.1 Overall subunit distribution

We restricted our study to the middle part of the forebrain, reaching from the striatum/pallidum to the amygdala (Block B in Figure 1). It included the parietal cortex, the striatum/pallidum, thalamus, amygdala, and the hippocampal formation. Like in rodents, we observed a wide and rather heterogeneous distribution of all investigated GABA_A_ receptor subunits. In general, labeling for subunits α1, β2, β3, and γ2 was especially prominent and abundant, that for subunits α2, α3, α5, β1, and δ appeared somewhat weaker; labeling was weakest for α4. We saw pronounced and often uniform labeling of dendritic areas, whereas we only rarely observed labeled perikarya. No obvious labeling was apparent for axonal tracts like the mossy fibers of the dentate gyrus.

It is, however, important to point out that labeling for each subunit depended on the avidity of the respective antibody and, therefore, did not allow quantitative comparisons between the expressions of different subunits. Also, comparisons of the subunit expression of a given subunit between different brain areas have only a semiquantitative character. The evaluation given in Table 2 refers to relative subunit immunoreactivities in individual brain areas in comparison to the brain area, in which this subunit is expressed highest. It does not compare immunoreactivities between different subunits.

**TABLE 2:** Abbreviations

Abbr.	Region	Presented in figure
AcbC	Nucleus accumbens, core	4
AcbSh	Nucleus accumbens, shell	4
AM	Anteromedial thalamic nucleus	8 and 9
AStr	Amygdalostriatal transition area	10
AV	Anteroventral thalamic nucleus	8 and 9
BLI	Basolateral amygdala, intermediate part	10
BLV	Basolateral amygdala, ventral part	10
BM	Basomedial amygdala	10
CA1	Cornu ammonis, Field 1	11
CA2	Cornu ammonis, Field 2	11
CA3	Cornu ammonis, Field 3	11
CA4	Cornu ammonis, Field 4	11
Cc	Corpus callosum	4 and 5
Cd	Caudate nucleus	4–6 and 8
Ce	Central amygdala	10
CeL	Central amygdala, lateral	10
CeM	Central amygdala, medial	10
CG	Cingulate gyrus	6
Cl	Claustrum	4–6
DB	Diagonal band	4
ECx	Entorhinal cortex	5 and 10
EGP	External globus pallidus	6
f	Fornix	9
Hip	Hippocampus	5
ic	Internal capsule	4, 6, and 8
ICC	Intercalated cell cluster	10
IGP	Internal globus pallidus	6
La	Lateral amygdala	10
LHb	Lateral habenular nucleus	9
MD	Mediodorsal thalamic nucleus	5
MDC	Mediodorsal thalamic n., central part	9
MDL	Mediodorsal thalamic n., lateral part	9
MDM	Mediodorsal thalamic n., medial part	9
Me	Medial amygdala	10
ML	Molecular layer of the dentate gyrus	11
MS	Medial septal nucleus	4
opt	Optic tract	5 and 6
PaS	Parasubiculum	11
Pir	Piriform cortex	4
PrCx	Perirhinal cortex	10
PrS	Presubiculum	11
Pu	Putamen	4–6
Rt	Reticular thalamic nucleus	5, 6, and 8
S	Subiculum	11
SNR	Substantia nigra, pars reticulata	7
st	Stria terminalis	6 and 8
STh	Subthalamic nucleus	7
TH	Temporal area TH (ECx, PrCx)	11
Thal	Thalamic nuclei	7
Tu	Olfactory tubercle	4
VAL	Ventral anterior thalamic n., lateral part	9
VAM	Ventral anterior thalamic n., medial part	8 and 9
VCo	Ventral cortical amygdaloid nucleus	10
VLL	Ventral lateral thalamic n., lateral part	5 and 9
VLM	Ventral lateral thalamic n., medial part	9
VMH	Ventromedial hypothalamic nucleus	6
VP	Ventral pallidum	4
VPM	Ventral posteromedial thalamic nucleus	9
VTA	Ventral tegmental area	7
ZI	Zona incerta	7
GAD67	Glutamate decarboxylase 67	2 and 4–11
NeuN	Neuronal nuclei/Fox‐3	2, 6, and 9–11

### 3.2 Parietal cortex

**FIGURE 3:**
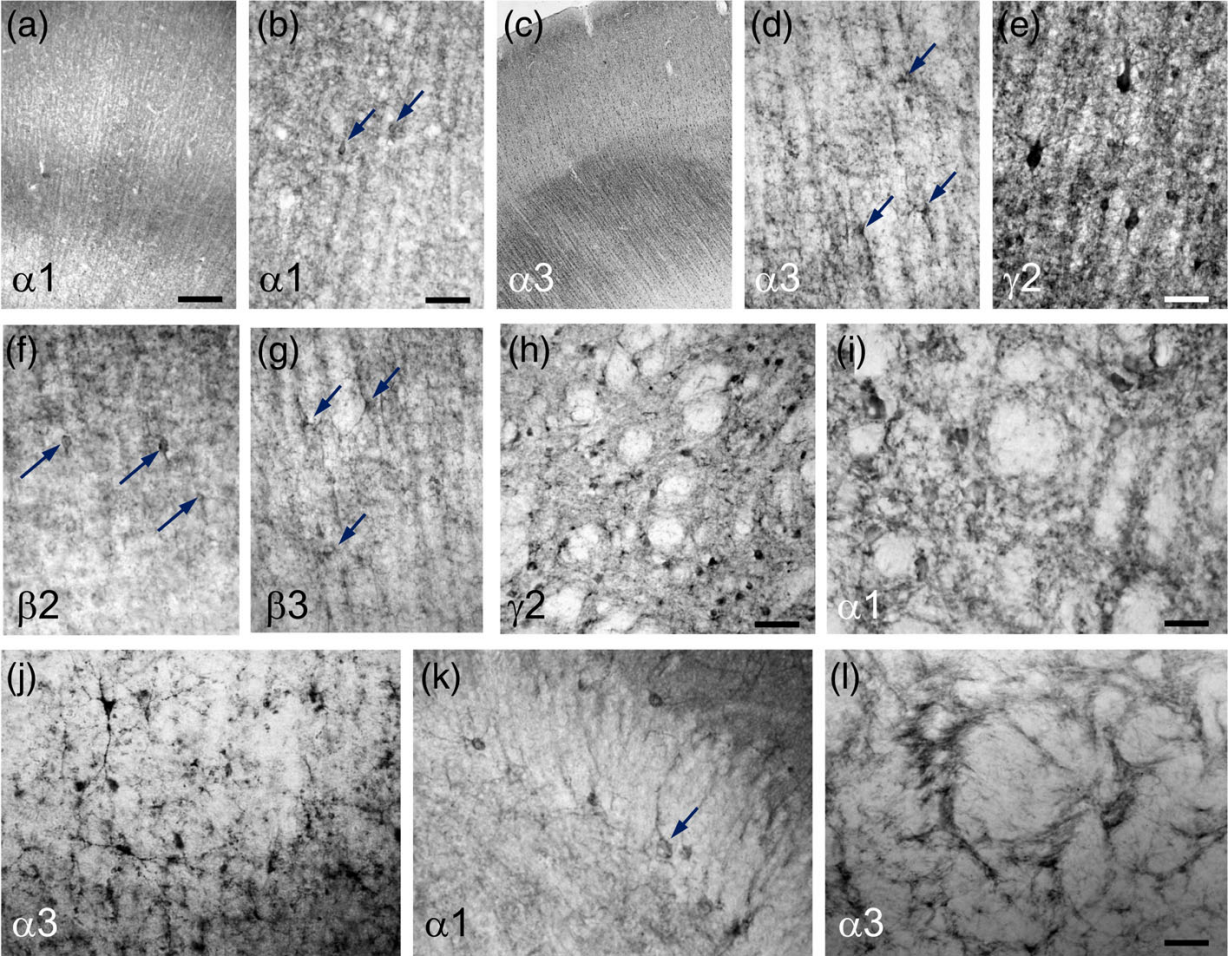
High magnification of GABA_A_ receptor subunit‐containing brain areas. The figure shows examples of GABA_A_ receptor subunits in individual neurons: α1‐subunit‐IR is shown throughout the cortical layers in (a), and at higher magnification in (b). It labels the dendritic field of pyramidal neurons; also single interneurons are labeled (arrows in b). α3‐IR is shown throughout the cortical layers in (c) and, at higher magnification in (d) showing small neurons. (e–g) γ2, β2, and β3 expressing neurons in the cortex are shown. (h,i) γ2‐ and α1‐IR in the caudate nucleus and in the subthalamic nucleus, respectively. (j,l) α3‐IR is shown in the deep cortex (border of Layer 6 and white matter) and the striatum, respectively. (k) α1‐IR is highly expressed in basket cells of the dentate gyrus (arrow). Arrows indicate neurons. Scale bar in a for (a and c), 400 μm; in (b) for (b and d), 200 μm; in (e) for (e–g), 100 μm; (h), 200 μm, in (i and l) for (i–l), 100 μm [Color figure can be viewed at wileyonlinelibrary.com]

Figure 2 shows the distribution of 10 GABA_A_ receptor subunit‐IR, of GAD67‐IR, and of NeuN‐IR in layers of the parietal cortex in coronal sections at the intraaural (Ia) level + 6.5 (parietal area, opercular part). We observed a strikingly heterogeneous expression of the individual subunits in different cortical layers. Subunits α1, α4, β2, β3, and δ were almost equally distributed throughout all cortical layers, α1, β2, and β3 at high concentrations, α4 and δ only at a much lower concentration (Figures 2 and 3, Table 3). Subunits β2 and β3 were, however, specifically enriched in a narrow band corresponding to Layer 4 whereas β1 was less expressed there. Subunits α2, α3, and β1 were more concentrated in the outer layers (Layers 1 and 2) and in the inner layers (5 and 6), whereas α5‐subunit prevailed in Layer 5 of the cortex (see also Figure 3a–f). We detected especially strong labeling for the γ2‐subunit in layers one to four. Subunit γ2 was considerably less abundant in Layers 5 and 6 and in the inner part of Layer 3, where labeling for subunit δ was marginally increased. Subunit α1, α2, α3, β2, β3, and γ2‐immunoreactive neurons were also seen in the deep cortical layers (white matter; Figure 3f,g,j). The GABA synthesizing enzyme GAD67 (contained in GABA neurons and their axons) was observed in all layers of the cortex but was enriched in layers two and four and thus followed the highest densities of nuclear cell labeling for NeuN. These labeling patterns were similar also in other parts of the cerebral cortex. Thus also at a more frontal level (Ia: 10) shown in Figure 5, the labeling patterns of cortical layers were similar as shown in Figure 2 (Ia: 6.5). Note for example the preferential labeling of the outer and inner layers by subunit α3 (and to a lesser extent) by α2, and preferential labeling of the inner layers by α5.

**TABLE 3:** Distribution and semiquantitative evaluation of GABA_A_ subunits. The expression of individual antibodies was evaluated in relation to the area of the highest expression of the respective antibody.

Region	Abbr.	Fig.	α1	α2	α3	α4	α5	β1	β2	β3		γ2	δ
*Cerebral cortex*	Cx	2–5											
Layer 1			+++	++	++	+++	++	+++	+++	+++		++	++
Layer 2			+++	+	++	+++	++	+++	+++	+++		+++	++
Layer 3			+++	+	+	+++	++	+++	+++	++		+++	++
Layer 4			+++		+	+++	+++	+	+++	+++		+++	++
Layer 5			++		+++	+++	++	+++	+++	++		++	+++
Layer 6			+++	++	+++	+++	++	+++	+++	+++		++	++
White matter			+					+	+				
Piriform cortex	Pir	4	+++	+++	+++	++	+++	+++	++	+++		++	++
Entorhinal cx	ECx	10	+++	+++	+++	+	+++	++	+++	++		+++	+
Perirhinal cx	PrCx	10	+++	+++	+++	+	++	++	+++	++		+++	++
Claustrum	Cl	4–6	+++	+	+++	+	++	+	++	+++		+++	++
Medial septum	MS	4	++	+	++	++	+	++	++	++		+	+
Tuberculum olfactorium	Tu	4	+++	+	+++	++	+++	++	+	+++		+	++
*Basal ganglia*													
Caudate nucleus	Cd	4–6	+++	+++	++	+++	+++	+++	+++	+++		++	+++
Putamen	Pu	4–6	+++	++	++	+++	++	++	++	++		++	+++
Globus pallidus, external	EGP	6	++	+	+	++	++	++	+	+		++	++
Globus pallidus, internal	IGP	6	+	+	+	+	+	+	+			++	++
Ventral pallidum	VP	4	++	++	+	++	+++	+	+	++		++	+
Diagonal band, Broca	DB	4	+++		++	+	+	+	+	++		+	+
Subthalamic nucleus	STh	7	++				+	++	+	+		+	+
Substantia nigra reticulata	SNR	7	+				++	+	++			++	+
Zona incerta	ZI	7	+				+	+	++			+	+
Accumbens, core	AcbC	4	++	++	+	++	+++	++	+	++		++	++
Accumbens, shell	AcbSh	4	++	++	++	++	+++	++	+	++		++	++
*Thalamus*													
Reticular nucleus	Rt	5,6,8	++	+	++	++	+	++	+			+	++
Mediodorsal thal. n													
Central part	MDC	9	+++	+	++	++	+	+	++	++		++	+
Medial part	MDM	9	+++	+	++	++	+	+	++	++		++	+
Lateral part	MDL	9	+++	+	++	++	+	+	++	++		++	+
Ventral lateral thal. nucleus													
Medial part	VLM	9	+++	+	++	+	+	+	+++	++		++	+
Lateral part	VLL	9	+++	++	++	++	+	+	++	++		++	+
Ventroanterior thal. nucleus													
Lateral part	VAL	9	+++	+	++	++	+	++	+++	++		++	+
Medial part	VAM	8 and 9	++	+	++	+	+	+	+	+		++	+
Ventral posterior thal n., medial part	VPM	9	++	+	+	++	++	+	+	+		++	+
Habenula, lateral	LHb	9	+	+	++	++	+	++	+	+		+	+
*Amygdala*													
Central amygdala	Ce	10	+	++	+	+		+		+		+	+
Basomedial amygdala	BM	10	++	++	+			++	+	++		++	+
Basolateral amygdala	BL	10	++	++	+	+	++	++	+	++		+	++
Lateral amygdala	La	10	+++	+++	+	+	+	+++	+++	+++		+++	++
Medial amygdala	Me	10	+	+	+			+		+		+	+
Cortical amygdala	VCo	10	++	+++	+		+	+++	+	++		+++	++
Intercalated cell cluster	ICC	10	+	+	++			+					+
*Hippocampus*													
Dentate gyrus													
Molecularlayer	ML	5,11	+++	+++			+++	+	++	+++		+++	++
CA4/Hilus	H	5,11	++	++		+		++		+		++	+
CA1	CA1	5,11	+++	+	+++	++	+++	++	+++	+++		++	+
CA2	CA2	5,11	++	++	+	+	+++	+++	+	++		++	+
CA3	CA3	5,11	++	++		+	+	+++		+		++	++
Subiculum	S	5,11	++		+	+	+	++	+++	+++		++	++
Presubiculum	PaS	5,11	++					+++	+++	+		+++	++
Parasubiculum	PrS	5,11	+++	+	+			+++	+++	++		+++	++

*Note:* +++, high; ++, medium; +, low; empty box, no labeling was detected. This rating refers to relative subunit immunoreactivities in individual brain areas (mainly representing dendritic fields) in comparison to the brain area in which this subunit is expressed highest. It does not compare immunoreactivities between different subunits.

### 3.3 Striatum, claustrum, and nucleus accumbens

**FIGURE 4:**
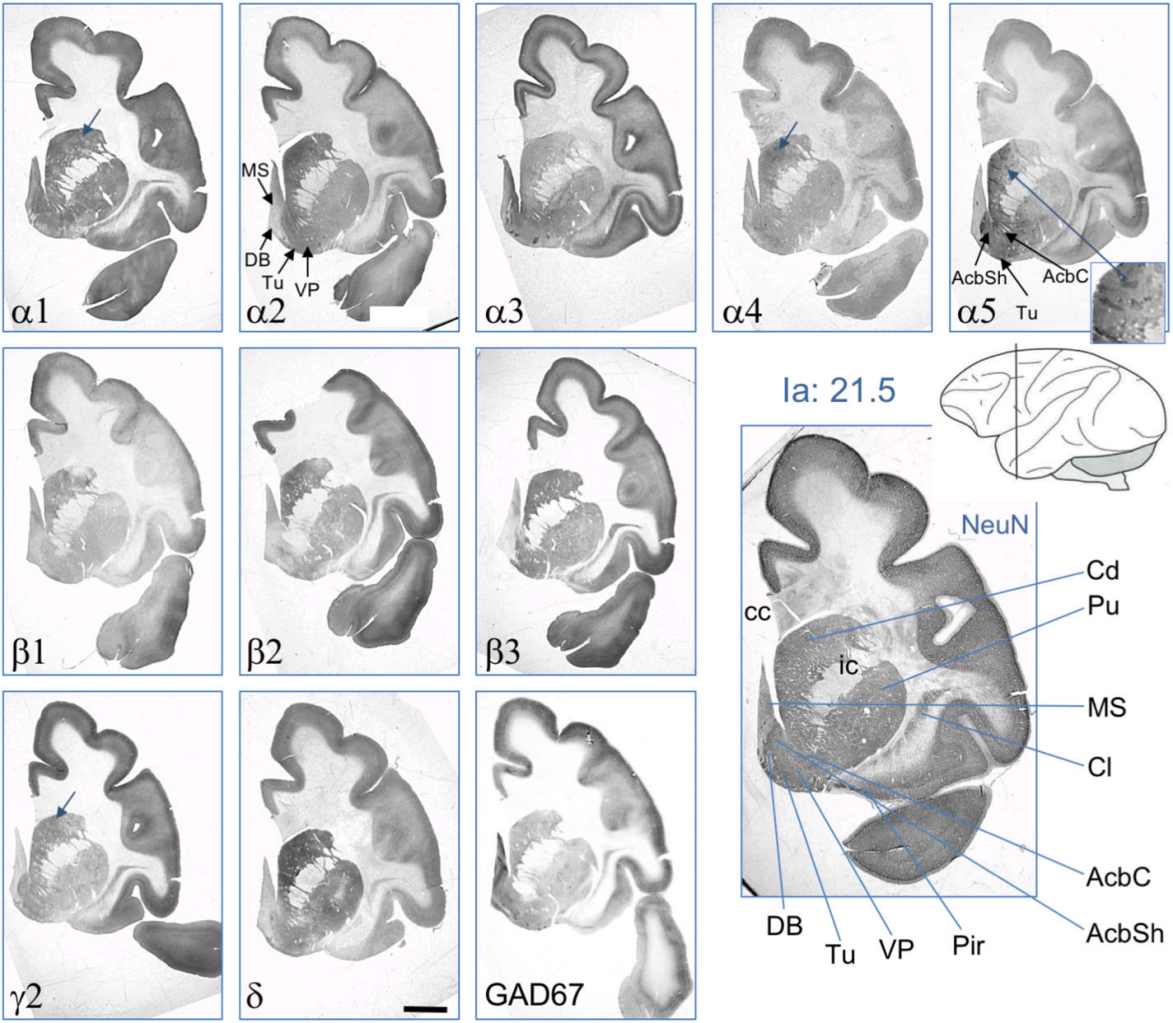
Expression of GABA_A_ receptor subunits at the level of the rostral striatum at Ia = 21.5, according to Paxinos et al. (2009). The insert at the α5 image shows a higher magnification of the striatum highlighting α5‐positive striosomes. Arrows denote possible striosomes. See also panels for α2 and α5 for anatomical details. For abbreviations, see Table 2. Scale bar = 3 mm [Color figure can be viewed at wileyonlinelibrary.com]

**FIGURE 5:**
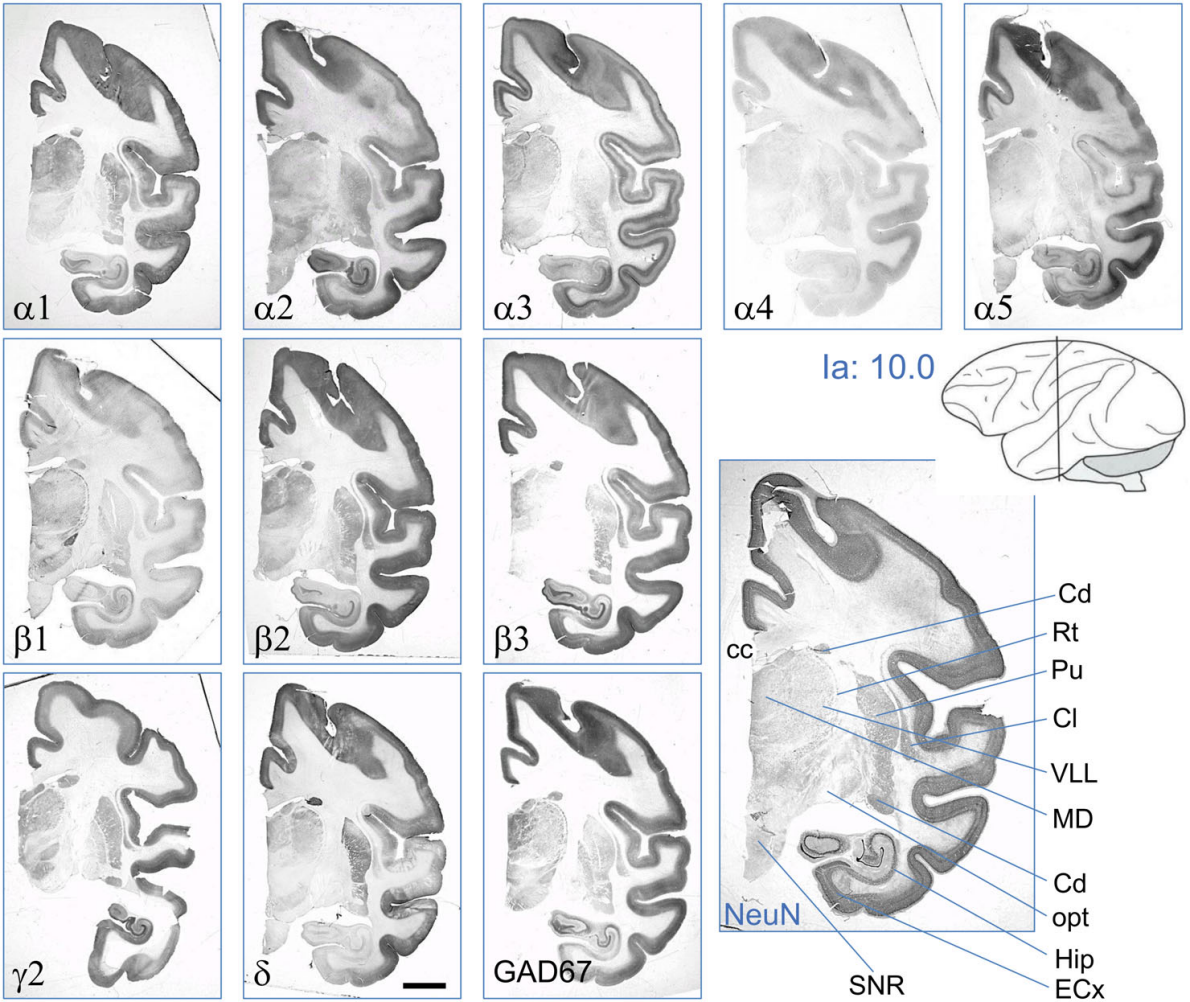
Distribution of GABA_A_ receptor subunits at the level of the caudate/putamen, the thalamic nuclei, and the rostral extension of the hippocampus. Depicted are serial sections at Ia = 10.0 (Paxinos et al., 2009). Compare labeling of basal ganglia, thalamic nuclei, and rostral hippocampus with more detailed expression of these brain areas at more rostral levels depicted in Figures 6, 8, and 11, respectively. For abbreviations, see Table 2. Scale bar = 3 mm [Color figure can be viewed at wileyonlinelibrary.com]

**FIGURE 6:**
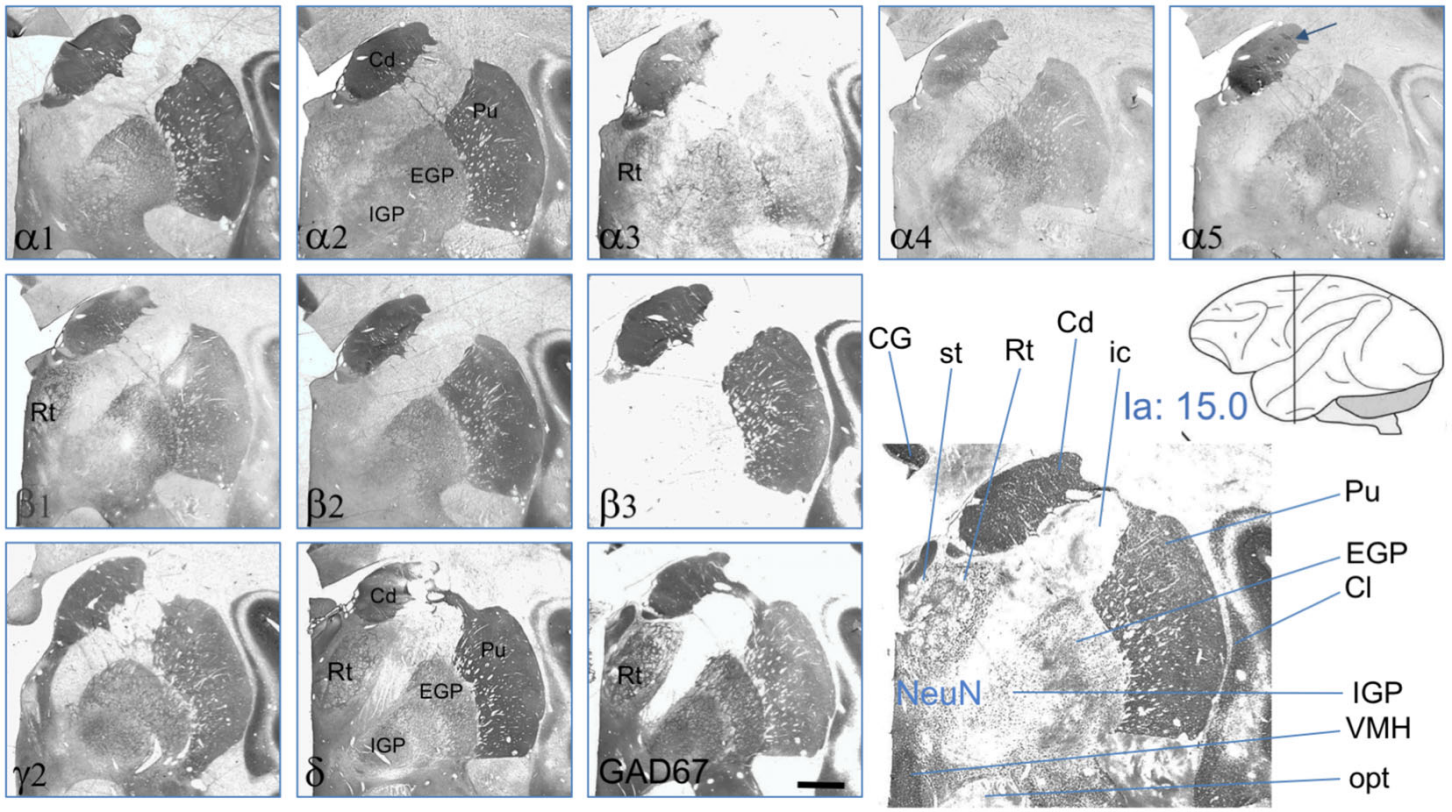
GABA_A_ receptor subunits at the caudal extension of the caudate/putamen and the globus pallidus in serial sections at the intraaural level Ia = 14.5–15 (Paxinos et al., 2009). Note that subunits α1, α2, β2, and δ are higher expressed in the striatum than in the pallidum and that subunits α4, α5, β1, and γ2 are roughly equally expressed in these areas. Subunit β3 is entirely absent in the pallidum, and (at this caudal level) subunits α3 and α5 are more expressed in the striatum than in the putamen. The arrow in the α5 image indicates α5‐positive striosomes. For abbreviations, see Table 2. Scale bar = 1.5 mm [Color figure can be viewed at wileyonlinelibrary.com]

Figures 4, 5, 6 show the distribution of GABA_A_ receptor subunits in the striatum (caudate nucleus and putamen), claustrum, ventral pallidum, and nucleus accumbens and allow comparing the labeling intensities in these areas with those in the overlying cortex. For all investigated subunits, we observed almost equal labeling in the caudate nucleus and the putamen, except for subunit α5, which was slightly enriched in the caudate versus the putamen. Compared with the overlaying cortical areas, labeling was about equally strong for subunits α1, α2, β1, β2, and β3 in the striatum and the cortex. Interestingly, in the caudate/putamen, we observed more intensive labeling for the δ‐subunit than for γ2 and even stronger than in the overlying cortex. Figure 3h,i shows well‐labeled α1 and γ2 positive neurons in the caudate. Like in the caudate/putamen, for all subunits, strong labeling was also found in the claustrum (Figures 4, 5, 6). We also observed a strong expression of most subunits (possibly except β2) in both subdivisions (core and shell) of the nucleus accumbens (Figure 4). Expression of α5 in both subdivisions was stronger than in the cortex or putamen. In the septum and the diagonal band, labeling was strong for α1, α3, and α4 (Figure 4). The ventral pallidum and tuberculum olfactorium were difficult to identify in our sections. It appeared that all subunits (possibly except β2 and δ) were present there (Figure 4). Notably, α5 subunit‐IR is enriched in striosomes (see insert in Figure 4) (Brimblecombe & Cragg, 2017). We suspect also similar patch‐like structures for other subunits (α1, α4, β3, and γ2), although these are by far not as clear as for α5 (Figure 4).

At a more caudal level (Ia: 15.0), we also observed similar labeling of the caudate and the putamen for most subunits (Figure 6). The caudate, however, was somewhat stronger labeled for the subunits α2, α3, and especially for α5 than the putamen. Labeling of the globus pallidus (external and internal segments) was generally lower than that in the caudate/putamen. Whereas the β3‐subunit was absent, subunit β1 was equally expressed as in the striatum. Compared to the major subunits α1 and α2, also subunits α4 and α5 were well recovered. Interestingly, subunits γ2 and δ revealed equally strong expression. The stria terminalis, which became visible in the same sections, was positive for α1, α2, α3, the β‐subunits, and γ2 (Figure 6).

### 3.4 Caudal putamen, entorhinal cortex

In Figure 5, aspects of the ventral putamen and the claustrum together with the external and internal segments of the globus pallidus are shown at level Ia 10. The figure also depicts the entire cerebral cortex at this level and aspects of the thalamus, the substantia nigra, and notably of the rostral parts of the hippocampus. Similar as at more rostral levels (Figure 4), also the caudal aspects of the caudate and putamen (Figures 5, 6) was rather strongly labeled for all subunits except for α3 and α5. The δ‐subunit was again clearly expressed and was even denser than γ2. Notably, subunits α1, α2, α5, β2, and δ were considerably higher expressed in the striatum than in the pallidum, whereas α4, β1, and γ2 were about equally expressed in the striatum and pallidum (Figure 6). As at the more rostral level at Ia 21 (Figure 4), subunit β3 was absent in the pallidum but well expressed in the striatum (Figure 6). All subunits (except α4 and δ) were highly expressed in the entorhinal cortex (Figure 5).

### 3.5 Substantia nigra and subthalamic nucleus

**FIGURE 7:**
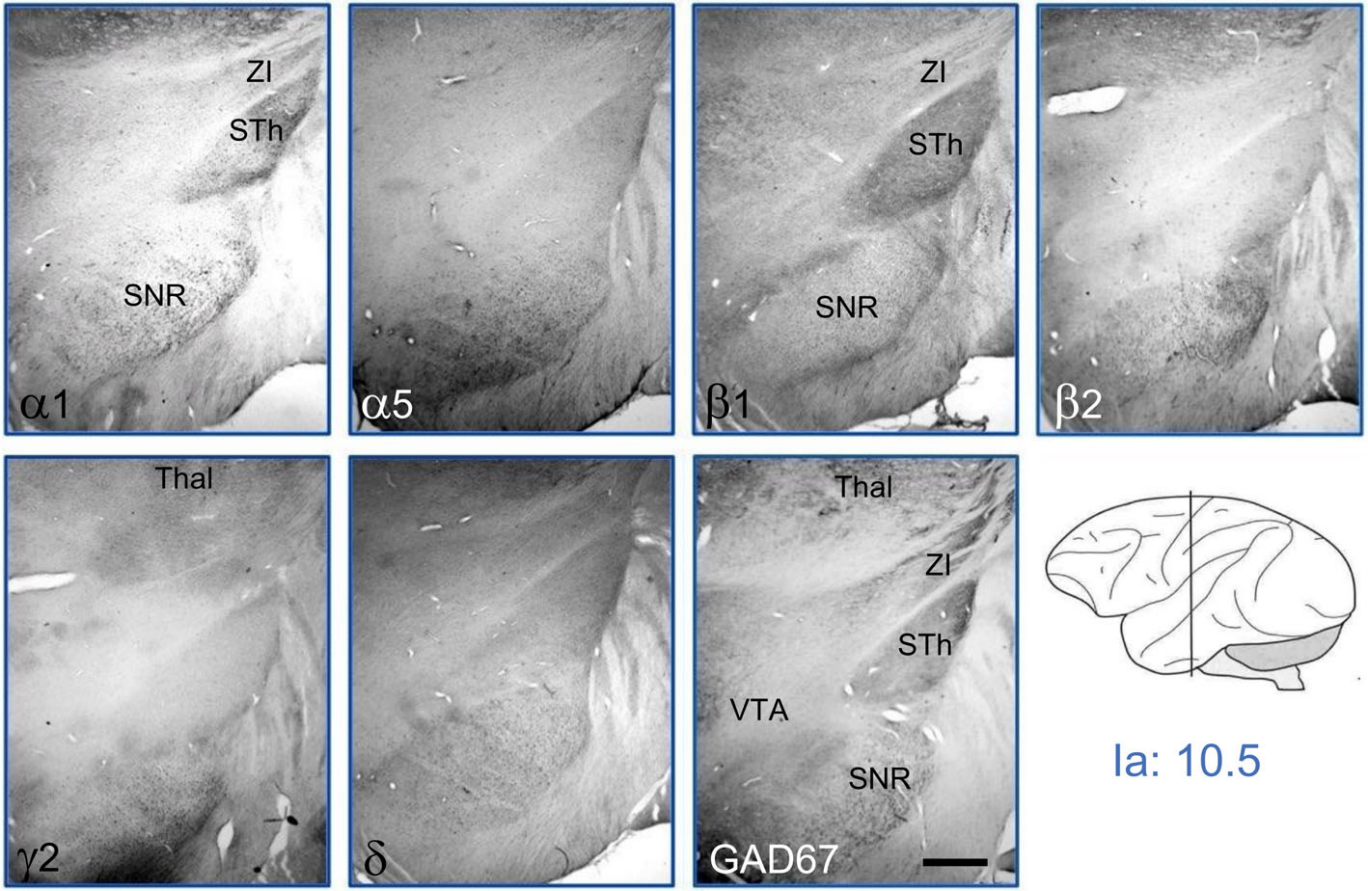
GABA_A_ receptor subunits in the substantia nigra, ventral tegmentum, and subthalamic nucleus at Ia = 10.5. There was low expression of α1, α5, β1, β2, γ2, and δ at the border of the substantia nigra reticulata. α1 and β1 were strongest in the subthalamic nucleus. We observed no significant labeling for subunits α2, α3, and α4 in this section. For abbreviations, see Table 2. Scale bar = 1.5 mm [Color figure can be viewed at wileyonlinelibrary.com]

In Figure 7, we show a detail at the level of the subthalamic nucleus, the substantia nigra, and the ventral tegmentum (Ia: 9.5–11). At this level, labeling for most subunits was weak. Faint expression was seen for subunits α1, α5, β1, β2, γ2, and δ. In the subthalamic nucleus, subunits α1 and β1 are expressed together with δ. In the substantia nigra pars reticulata, subunits α1, α5, and β2 are expressed together with β1 (at its borders), γ2, and δ. In the ventral tegmentum, faint labeling may also be present for these subunits. GAD67‐IR, however, clearly outlines the areas by labeling GABA containing neurons or nerve terminals.

### 3.6 Thalamus

**FIGURE 8:**
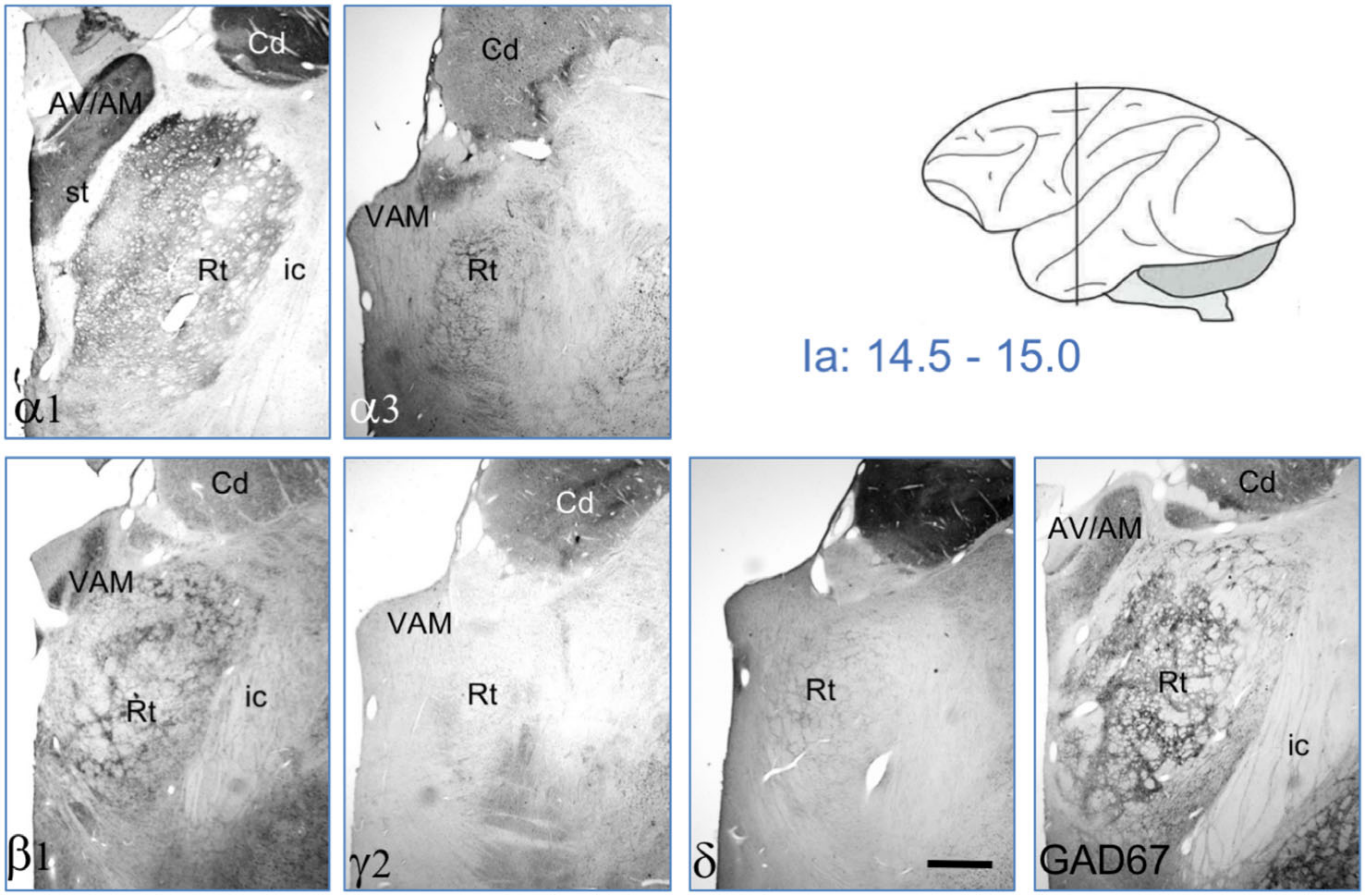
GABA_A_ receptor subunits in the reticular thalamic nucleus. Serial sections at Ia = 14.5–15.0 (Paxinos et al., 2009). The nucleus seems to express only α1, α3, β1, γ2, and δ containing GABA_A_ receptors. Note the high expression of GAD67 in the reticular nucleus. For abbreviations, see Table 2. Scale bar = 1.5 mm [Color figure can be viewed at wileyonlinelibrary.com]

**FIGURE 9:**
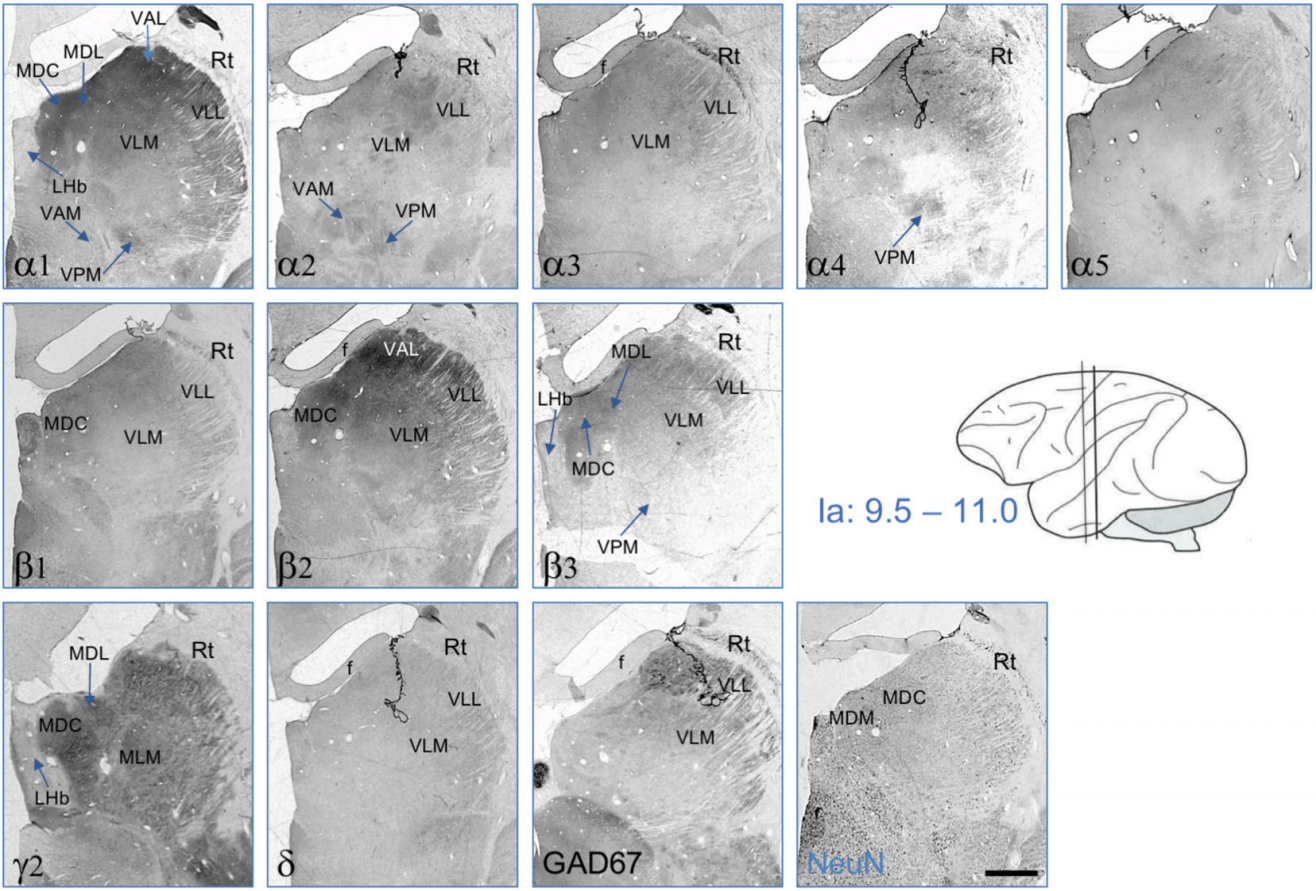
Distribution of GABA_A_ receptor subunits in nuclei of the dorsal thalamus. Serial sections at Ia = 9.5–11.0 (Paxinos et al., 2009). Note the abundant expression of most subunits in almost all nuclei. Subunit β1 and δ are, however, almost entirely absent and α3 and α5 expressed only at low levels. Scale bar = 1.5 mm [Color figure can be viewed at wileyonlinelibrary.com]

The reticular thalamic nucleus presents itself as a narrow band above the other thalamic nuclei around the intraaural level 9.5–11 (Figure 9 8) and at the more frontal level 14.5–15 as a broader formation with a typical network structure (Figures 6 and 8 5 and 7). The subunit labeling patterns were identical at both levels. There was strong staining for α1, α3, β1, and δ, whereas labeling for subunits α2, α4, α5, β3, and γ2 was either weak or absent (Figures 6, 8, and 9 5, 7, and 8, Table 2). The reticular thalamic nucleus contained also high concentrations of GAD67‐IR.

The dorsal thalamic nuclei showed entirely different labeling patterns (Figure 9, Table 2). In the mediodorsal thalamic nuclei (central, lateral, and medial aspects; MDC, MDL, MDM) we observed strongest labeling for subunits α1, α2, α4, β2, β3, and γ2, but also α3, α5, and δ were expressed although at low levels. In the ventrolateral nuclei (medial and lateral parts; VLM, VLL), again subunits α1, α2, β2, β3, and γ2 are strongest; in these parts of the thalamus, subunit α5 appeared to be more expressed than α4. Also the δ‐subunit was present although at a low level. We detected subunits α1, α2, and β3, together with α4, α5, γ2, and δ also in the ventral posteromedial thalamic nucleus. Taken together, in most thalamic nuclei (except the reticular thalamic nucleus) subunits α1, α2, β2, β3, and γ2 were expressed at high levels and α4, α5, and δ at considerably lower levels. The β1‐subunit was expressed in the mediodorsal, ventrolateral, and the ventral anterior and ventral posterior thalamic nuclei at moderate concentrations, and α3 was observed only in the ventrolateral and mediodorsal aspects of the thalamus and there at low concentrations. In the lateral habenular nucleus, most subunits appear to be expressed except for α1 and γ2 (Figure 9).

### 3.7 Amygdaloid nuclei

**FIGURE 10:**
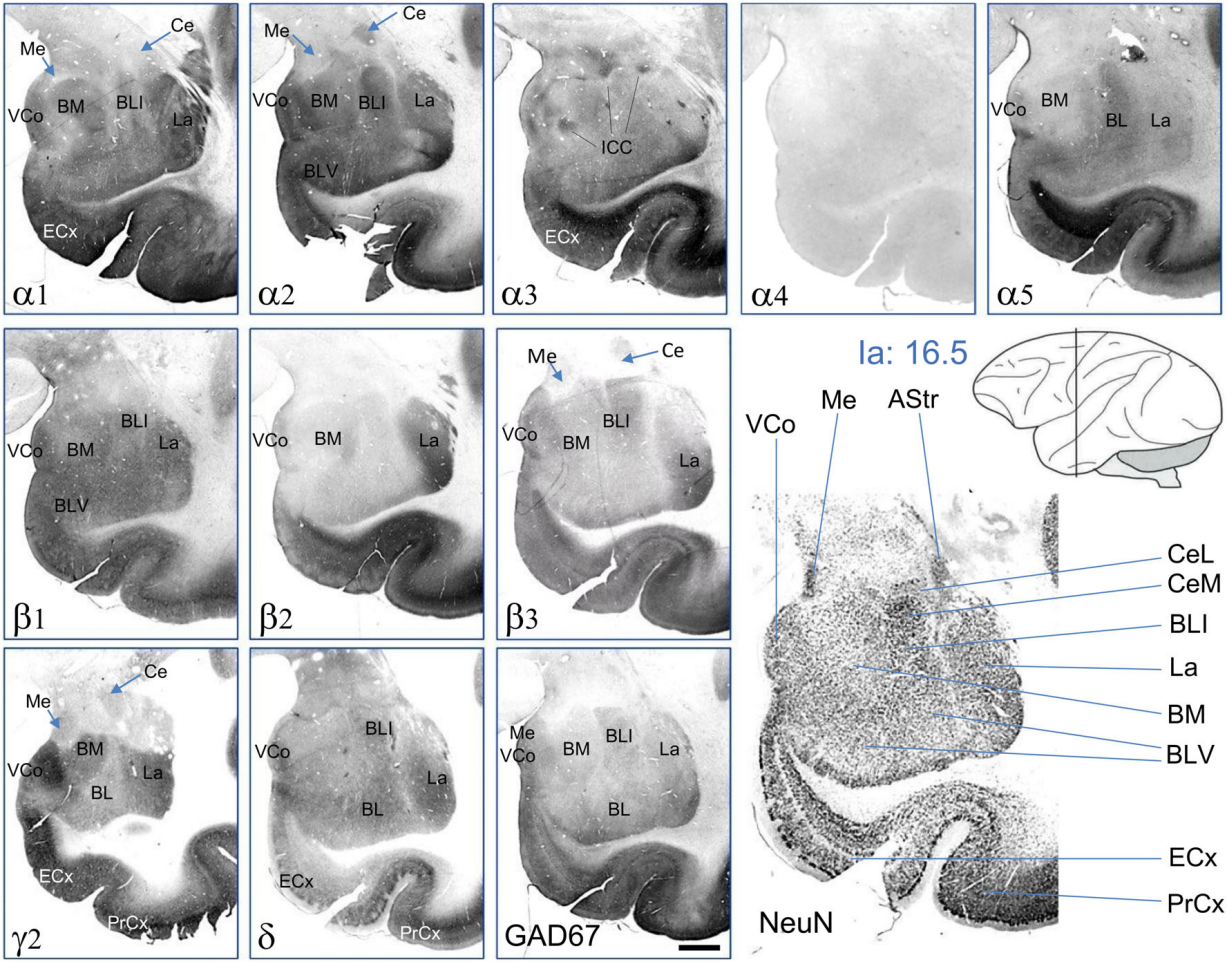
Distribution of GABA_A_ receptor subunits in amygdaloid nuclei. Serial sections were from Ia = 16.5. Note the strong expression and distinct distribution of most GABA_A_ receptor subunits in many amygdaloid nuclei, whereas α1, α2, β1, and γ2 were expressed in virtually all areas of the amygdala, β2 was restricted to the lateral amygdala (LA). Also α5, β3, and δ were restricted to the basal (BM) and basolateral nuclei (BL, BLI), and, most strikingly, α3‐IR labeled exclusively all intercalated cell clusters (ICC). Subunits α1, α2, α5, β1, β3, γ2, and δ are expressed in the cortical amygdala; α1, α2, β1, and γ2 are present in the medial (Me) and α2, β3, and γ2 in the central nucleus (Ce). For abbreviations, see Table 2. Scale bar = 1.5 mm [Color figure can be viewed at wileyonlinelibrary.com]

With the exception of α4, all GABA_A_ receptor subunits were expressed at varying, but mostly high levels throughout the amygdaloid nuclei (Figure 10). The δ‐subunit was observed throughout the amygdala, though at low concentrations. Strikingly, the α3‐subunit labeled all intercalated nuclei.

Whereas subunits α1, α2, α3, and γ2 were clearly expressed in the central amygdala (Ce), we did not observe significant concentrations of any subunit in the medial amygdala (Me) except for weak labeling for γ2 and δ and, to a lesser extent, for α1, α3, and β1. In the basomedial amygdala (BM) subunits α1, α2, α3, β1, β2, γ2, and some δ were contained. In the lateral amygdala (LA), significant subunit‐IR was found for α1, α2, β1, β2, β3, γ2, and δ, and at a lower level for α5. In the basolateral amygdala, including the intermediate and ventral aspects (BL, BLI, BLV) subunits α1, α2, α3, α5, β1, γ2, δ, and lesser β3 were observed. The cortical amygdala, notably the ventral cortical amygdala expressed subunits α1, α2, α3, β1, γ2, and δ at high concentrations and β3 at somewhat lower concentrations. We also observed high concentrations of all subunits (except α4, but including α3) in the entorhinal cortex. Subunits α3, α5, and β2 were especially concentrated in its inner layers, α2, β1, and γ2 in the outer layers and α1 and β3 throughout the area (Figure 10).

### 3.8 Hippocampus, subiculum, presubiculum, and parasubiculum

In the molecular layer of the dentate gyrus, subunits α1, α2, β2, β3, and γ2 were highly expressed, α5 only at low concentrations. Also subunits β1, α4, and δ were expressed, however, at very low concentrations. In the hippocampus proper, we observed subunits α1, α2, α3, β1 (not β2), β3, and γ2 in the stratum oriens of CA3 and subunits α1, α2, α3, α5, all β‐subunits, and γ2 in the stratum oriens of CA1. Immunoreactivities for α1, α3, α4, α5, β1, β2, β3, and γ2 extended to the strata radiatum and lacunosum moleculare of CA1 and were there strongest for α3, β3, and γ2. The profile of GABA_A_ receptor subunit expression in the subiculum, presubiculum, and parasubiculum was similar as in sector CA1. The expression levels, however, were lower for α2, α3, α5, and β3 in the presubiculum and parasubiculum than in CA1 and the subiculum. We observed δ‐subunit‐IR in all subregions of the hippocampal formation, although at rather low concentrations. Note that the high expression of GAD67 is within the excitatory (glutamatergic) mossy fiber pathway.

## 4 DISCUSSION

Our study is the first comprehensive analysis of the immunohistochemical distribution of 10 GABA_A_ receptor subunits (α1–α5, β1–β3, γ2, and δ) in the rhesus monkey forebrain. However, there are light and electron microscopic studies on the distribution of subunits α1 and β2/3 (using an antibody detecting these two subunits together) in the baboon (Waldvogel et al., 1998) and of subunits α1, α2, α3, β2/3, and γ2 in different brain areas and the spinal cord of humans including Huntington's Chorea, Alzheimer, and temporal lobe epilepsy patients (Kwakowsky et al., 2018; Loup et al., 2000; Pirker et al., 2003; Waldvogel et al., 1990; Waldvogel et al., 2008; Waldvogel et al., 2017; Waldvogel & Faull, 2015). In recent studies, we (Pirker et al., 2003) and Loup et al. (Loup et al., 2000) investigated the changes in the expression of subunits α1–α3, β1–3, and γ2 in the hippocampus of patients with temporal lobe epilepsy and in controls and Stefanits et al. (2018) studied the distribution of subunits α1, α2, α3, α5, β2, β2/3, and γ2 in the human hippocampus and amygdala.

### 4.1 Antibodies

In our present study, we investigated immunoreactivities for 10 GABA_A_ receptor subunits (α1–α5, β1–β3, γ2, and δ). We used purified rabbit antibodies against epitopes of the rat/mouse proteins. Except for α4, α5, and δ, the respective epitopes of monkey and human subunits are identical with the ones of the rat suggesting similar labeling of the subunits as in the rat. The peptide sequences used for generating rat α4‐, α5‐, and δ‐subunit antibodies differ to some extent from the amino acid sequences of the respective human protein segments (see Table 1). All antibodies have been characterized in the rat tissue by preadsorption to synthetic peptides previously (Sperk et al., 1997). Here we used synthetic peptides equivalent to the respective sequences of the monkey/human α5‐ and δ‐subunits to prove binding of these antibodies to the monkey receptor proteins. Both peptides (at a concentration of 1 μg/ml) were capable of preventing antibody binding to the respective receptor subunits in sections of the monkey brain, supporting the specificity of the labeling (data not shown). Furthermore, the heterogeneous distribution of individual subunits widely resembling that observed in the rat and mouse supports the specificity of all antibodies used (Hörtnagl et al., 2013; Pirker et al., 2000; Schwarzer et al., 2001; Sperk et al., 1997). Subunit α4, however, was less prominently labeled in the monkey than in the rat. This may be due to reduced avidity of the (rat) antibodies for the respective monkey epitopes or to lower expression levels of this subunit (this may also be considered for α5‐ and δ‐subunit antibodies).

### 4.2 Subunit distribution in the monkey compared to that in the rat and human

Whereas the studies in the rat and mouse addressed the distribution of a broad range of GABA_A_ receptor subunits (α1‐α6, β1‐β3, γ1, γ2, and δ) (Fritschy & Mohler, 1995; Hörtnagl et al., 2013; Pirker et al., 2000; Schwarzer et al., 2001; Sperk et al., 1997) the equivalent investigations in the human brain were mostly restricted to subunits α1, α2, α3, combined IR for β2 and β3, and subunit γ2 (Kwakowsky et al., 2018; Waldvogel et al., 1998; Waldvogel et al., 2008; Waldvogel et al., 2017). In a recent study on the hippocampus and amygdala, also β2 (besides β2/β3) and α5 were included (Stefanits et al., 2018). In Table 4, we present an overview comparing the distributions of the GABA_A_ receptor subunits in the rat and the monkey brain and discuss this as follows.

**TABLE 4:** Primary expression of GABA_A_ receptor subunits in the monkey compared with the rat. Note that the expression of GABA_A_ receptor subunits is more heterogeneous in the monkey than in the rat, where in most areas, their expression is more distinct and restricted to fewer subunits

Brain area	Rat		Monkey	
	Major subunits	Minor subunits	Major subunits	Minor subunits
**Parietal cortex**	α1, β2, β3, γ2		α1, β2, β3, γ2	
**Caudate/putamen**	α2, β3, γ2		**α1**, α2, α3, **α4, α5**, β1, **β2,** β3, γ2, **δ**	
**Globus pallidus**	α1, β2, γ2, γ1	α3, α4, α5	α1, **α2,** γ2, **δ**	
**Thalamic nuclei** (general)	α1, α4, β1, β2, δ	γ2	α1, **α2**, α4, β2, **β3**, γ2	α3, α5, δ
Reticular n.	α3, α4, β1, γ2		**α1,** α3, β1, γ2	δ
**Amygdala**				
Central	α1, α2, β1, β2, γ2, γ1		α2, **β3**	α1, γ2
Medial	α2, β1, β2, γ1	α1, β3, γ2	α1, α2	α1, γ2
Lateral	α1, α2, β1, β2, γ2		α1, α2, β1, β2, **β3**, γ2, **δ**	α5
Basomedial	α1, α2, β1, β2, γ2		α1, α2, β1, γ2	
Basolateral	α1, α2, β1, β2, γ2		α1, α2, **α5**, β1, **δ**	β3, γ2
Cortical	α1, α2, β1, β2, γ2		α1, α2, **α5,** β1**, β3**, γ2, **δ**	
Intercalated cell cluster			α3	
**Hippocampus**				
Dentate molecular layer	α2, α4, β1, β3, γ2, δ		**α1,** α2, β3, γ2	α4, α5, β2, δ
CA3	α5, β3, γ2		**α1, α2,** β1, γ2	
CA1	α5, β3, γ2		**α1, α2,** α3, α5, **β1, β2,** β3, γ2	
Subiculum	α1, β1, β2, β3, γ2		α1, α2, β1, γ2	

*Note:* Subunits shown in bold indicate subunits that are expressed in the monkey in addition to the rat.

#### 4.2.1 Cortex

Like in the rat, (Drexel, Kirchmair, & Sperk, 2013; Fritschy & Mohler, 1995; Pirker et al., 2000; Tsunashima et al., 1997; Wisden et al., 1992) subunits α1, β2, β3, and γ2 were expressed highest in the monkey cortex. Subunit α4 is only faintly present in the monkey cortex, whereas it is well expressed in the outer cortical layers of the rat. In contrast, subunit α5 is clearly expressed in the monkey but not in the rat cortex. Hendry et al. (1992) investigated the expression of subunits α1, β2/β3, and γ2 in the macaque and human visual cortex. For all three subunits, they observed the highest expression in Layers IVA and IVC and Layers II to III being in accordance with our present results in the monkey. Also, Waldvogel et al. (2017) reported strong labeling for α1 (in addition to α2), β2/3, and γ2 (strongest in Layer 4) but not for α3 in the human parietal cortex.

#### 4.2.2 Basal ganglia

In our recent study on the distribution of 13 different subunits in the basal ganglia of the rat, one of the most striking results was the high expression of subunits α2 and β3 in the striatum opposed by low expression in the pallidum (Pirker et al., 2000). In reverse, subunits α1 and β2 were highly expressed in the globus pallidus of the rat but were low in the striatum. The δ‐subunit was only expressed in the striatum, not in the pallidum and, in reverse, γ2 and γ1 were present in the pallidum but not in the striatum. In the rat, subunits α4 and α5 were also expressed in the striatum (although at considerably lower levels than α2), but not in the globus pallidus.

In the macaque, these differences between striatum and pallidum and expression of subunits α2/β3 and α1/β2, however, are by far not that distinct (see Figure 6; Table 4). In the monkey, we observed expression of all subunits in the striatum (caudate and putamen). In contrast to the rat, labeling for α1 and α2 appears to be equally strong in the striatum and, at a lower concentration, also in the pallidum. In the striatum of the macaque, also subunits α3, α4, and α5 are expressed at concentrations presumably comparable to those of α1 and α2. Expression of these subunits is, however, lower in the pallidum than in the striatum. In contrast to the rat, all three β‐subunits are expressed in the caudate/putamen. Strikingly, subunit β3 is entirely absent both in the rat and monkey pallidum, and (other than in the rat where δ was dominating in the striatum and γ2 in the pallidum) we observed equal expression of subunits γ2 and δ both in the striatum and (at a lower level) in the pallidum of the monkey. Taking into account a possibly lower avidity of the δ‐subunit antibody to the macaque protein, expression of the δ‐subunit may even prevail that of γ2.

In their initial study on the distribution of α1‐ (bd24‐) and bd17‐IR (labeling β2/β3 subunits), Waldvogel et al. (1998) reported strong labeling of the caudate/putamen and (lesser) of the pallidum for α1 and high β2/β3‐IR in the striatum and considerably less in the putamen (Waldvogel et al., 1998). In their recent study, they reported an equal, apparently low expression of subunit α1 in the striatum and pallidum and a rather high expression of α2 in the caudate and putamen, which was by far exceeding that in the pallidum and was consistent with our rat data and our present data on α2 in the monkey (Waldvogel et al., 2017). They observed comparatively low expression of subunit γ2 in the striatum and putamen suggesting possible expression of δ‐ and even γ1‐subunits that was not included in their study.

#### 4.2.3 Thalamus

In the reticular thalamic nucleus, we observed principal labeling for subunits α1, α3, β1, and γ2 in the monkey, whereas in the rat subunits α3, α4, β1, and γ2 are dominating (Drexel et al., 2015; Pirker et al., 2000).

In the majority of all other thalamic nuclei of the monkey, we observed strong expression of α1, α2, α4, β2, β3, and γ2, whereas in the rat, the majority of the thalamic nuclei were positive for subunits α1, α4, β1, β2, and δ (Table 4). Expression of subunits α3 and δ was less prominent in the monkey than in the rat and confined to the ventrolateral nuclei; expression of α5 appears to be stronger in the macaque than in the rat. In the dorsal thalamus of the rat, also expression of β2 was considerably stronger than that of β3, whereas it is about equal in the monkey. In most thalamic nuclei of the monkey, we observed in addition expression of subunits α4 and α5, which was about equal to that of α3.

Our monkey data are in agreement with those by Waldvogel et al. in the human thalamus (Waldvogel et al., 2017). They observed considerable expression of all investigated subunits (α1, α3, β2/β3, and γ2) but less for α2. In the reticular thalamic nucleus, they also observed strongest labeling for α3 and γ2. Taken together, there are some significant differences in the distribution of GABA_A_ receptors in the thalamus between rat and monkey. Whereas subunits α1, α4, β2, and δ are by far prevailing in the rat thalamus (Drexel et al., 2015; Pirker et al., 2000), subunits α1, α2, α4, β2, and equally β3 and γ2 are highest expressed in the monkey. Subunit δ is less and α5 more prominent in the monkey thalamus than in the rat.

#### 4.2.4 Amygdala

In the amygdala of the rat subunits α1, α2, β1, β2, β3, and γ2 are most abundant (Fritschy & Mohler, 1995; Pirker et al., 2000). We also observed expression of subunit γ1 in the central and medial nuclei of the rat (Pirker et al., 2000). In the amygdala of the macaque, the subunit distribution is even more distinct than in the rat (see Figure 10): Subunits α1, α2, and γ2 are most widely distributed, although α1 and γ2 are present only at low concentrations in the central and medial nuclei; in contrast, α2 is especially well expressed in the central nucleus. Subunits α2, α1 and β1 are expressed about equally in the basomedial, basolateral, and lateral nuclei, whereas α5, β3, and δ are mainly expressed in the lateral and basolateral (not in the basomedial) nuclei. Subunit β2 appears to be restricted to the lateral nucleus, and α4 is almost not expressed in any nucleus of the amygdala. The cortical amygdala expresses α1, α2, α5, β1, β3, γ2, and δ. Also the distribution of subunits α1, α2, α3, α5, and β2 (besides β2/β3) studied by Stefanits et al. (2018) in the human amygdala showed similar results as ours in the macaque. Thus they reported the strongest labeling for α1 in the LA, followed by labeling of the cortical, medial, central, and basomedial nuclei and lower staining in the basolateral amygdala. Subunit α5 was higher in the cortical and basomedial parts than in other nuclei. β2 and β2/ β3 were enriched in the lateral compared to the basolateral nucleus. Like in the monkey brain (and other than in the rat), subunit α3 was almost confined to the intercalated amygdaloid nuclei.

##### Hippocampus

**FIGURE 11:**
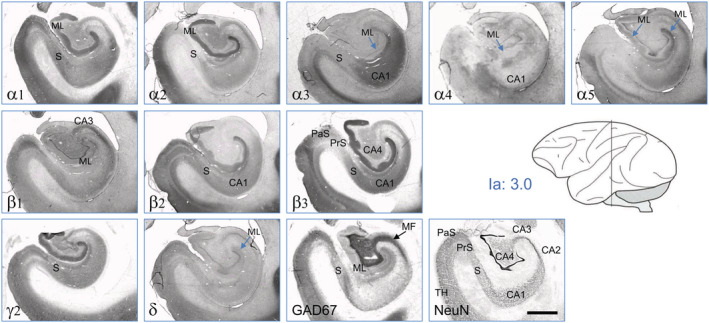
GABA_A_ receptor subunits in the hippocampus, subiculum, presubiculum and parasubiculum. Serial sections at intraaural level = 3.0. Subunits α1, α2, α5, β2, β3, γ2, and (less) δ are expressed in the molecular layer of the dentate gyrus (ML). α1, α2, α3, α4, α5, β1‐β3, γ2, and δ are expressed throughout the Ammon's horn (α1, β2, and β3 are less expressed in CA3). Most subunits are present in the subiculum (S), presubiculum (PrS), and parasubiculum (PaS) (there except α2 and β3). For abbreviations, see Table 2. Scale bar = 1.5 mm [Color figure can be viewed at wileyonlinelibrary.com]

In the molecular layer of the rat hippocampus, primarily subunits are α2, α4, β1, β3, γ2, and δ whereas in the Ammon's horn, subunits α5, β3, and γ2 are the most abundant ones (Fritschy & Mohler, 1995; Pirker et al., 2000; Sperk et al., 1997). In contrast, α1, α2, β3, and γ2, followed by α5, β2, and δ are the prevailing subunits in the monkey dentate molecular layer. In sector CA3 and in the subiculum of the monkey, α1, α2, β1, and γ2 are predominating, whereas α1, α2, α3, α5, all β‐ and γ2 are strongest in sector CA1 (see Figure 11, Table 4) In contrast, in the Ammon's horn of the rat, subunits α5, β3, and γ2 are most abundant (Pirker et al., 2000; Sperk et al., 1997).

Our data in the monkey are generally in agreement with the study by Stefanits et al. (2018) on the human hippocampus. Both in the monkey and in humans, subunits α1 and α2 are about equally expressed in the Ammon's horn and in the molecular layer of the dentate gyrus, and expression of α3, α5, and β3 (β2/β3 in the human) are considerably higher in sector CA1 and the subiculum than in the other hippocampal areas (CA3, dentate gyrus) of human and monkey hippocampus. Also subunit γ2 is rather low in the human compared to the monkey and as reported in recent studies on the human hippocampus (Kwakowsky et al., 2018; Pirker et al., 2003). Again, like in the striatum, expression of GABA_A_ receptor subunits is less distinct in the monkey and human than in the rat. Thus, the clear difference in the dominant expression of subunits α2, α4, and β3 in the dentate gyrus and of α5 in the Ammon's horn (CA1–CA3) of the rat is opposed by a broad subunit expression in macaque and human (α1, α2, α5, all β‐subunits in the dentate and α1, α2, α3, α5, all β‐subunits in CA3).

### 4.3 Functional aspects

The most striking finding of our present study is the considerably broader presentation of the majority of GABA_A_ receptor subunits in the macaque brain compared to the rat. Thus, we did not observe the distinct differences in the expression patterns seen, for example, in the rat basal ganglia (expression of subunits α2/β3 vs. α1/β2 in the striatum and pallidum, respectively) or in the hippocampal formation (α2/α4/β3/δ vs. α5/β3/γ2 in the dentate gyrus and sector CA1, respectively) to a similar extent in the monkey brain. Instead, many brain areas (e.g., thalamus, amygdala, also hippocampus) expressed many subunits almost uniformly in all subfields. On the other hand, layer‐specific expression of different subunits was still seen, for example, in the parietal cortex.

In general, most of the GABA_A_ receptors consist of two α‐, two β‐, and one γ‐ or δ‐subunit. The binding pocket for GABA is located at the interface of one α‐ with a neighboring β‐subunit, and the benzodiazepine sensitivity is mediated by an α‐ (α1, α2, α3, or α5) together with the γ2‐subunit. Receptors containing either α4‐, α6‐, γ1‐, or δ‐subunits or receptors containing only α‐ and β‐subunits do not respond to benzodiazepines (Gunnersen, Kaufman, & Skolnick, 1996; Sieghart & Savić, 2018). Instead, α1/β3‐containing receptors are sensitive to stimulation by neurosteroids (Wang, 2011). Furthermore, δ‐subunit containing GABA_A_ receptors (often in combination with α4 or α5) exert a high affinity to GABA and GABA agonists (Mertens, Benke, & Mohler, 1993), which makes them privileged for being still active at sites remote from the GABA terminal. In the rat, these extrasynaptically or perisynaptically located receptors often mediate tonic inhibition, whereas α1‐, α2‐, α3‐, and β‐ and γ2‐containing receptors (located within the synapse) transmit phasic inhibition (Mody, 2001). If this holds for primates or the human has still to be determined.

Taken from their regional distribution, the majority of GABA_A_ receptors in the monkey contains γ2 and an α‐subunit responding to benzodiazepines and may be capable of mediating phasic inhibition. The extrasynaptically located δ‐subunit containing GABA_A_ receptors are especially rich in the cerebral cortex, in the basal ganglia and amygdala (notably not in thalamic nuclei), suggesting that they mediate tonic inhibition in these brain areas. The concentrations of GABA_A_ receptors (containing subunits α1, α2, α3, α5, β1, β2, β3, and γ2) in the cortex and amygdala are consistent with the sedative and anxiolytic actions exerted by benzodiazepines and neurosteroids. The high concentrations of GABA_A_ receptors in the basal ganglia are intriguing. They are neither reflected by actions (or side effects) of benzodiazepines, nor of neurosteroids. GABA released from interneurons in the striatum upon other interneurons or GABA neurons projecting to the pallidum are acting on δ‐subunit containing (possibly extrasynaptic) receptors and may mediate tonic inhibition upon these neurons. The pallidum contains equally δ‐ and γ2‐subunit‐containing receptors suggesting both phasic and tonic inhibition. They may be located either on interneurons or on GABA‐ergic neurons projecting to the substantia nigra and may, thus, mediate phasic and/or tonic inhibition. Also the reticular thalamic nucleus giving rise to GABA‐ergic projections contains both γ2‐ and δ‐subunit containing receptors. In striking contrast, most nuclei of the dorsal thalamus of the monkey contain only the γ2‐subunit and no δ‐subunit. Thus, GABA_A_ receptors in dorsal thalamic nuclei (located on interneurons or glutamatergic projection neurons) may respond fast and more precisely to the synaptically released GABA by phasic inhibition. Also in the dentate gyrus expression of α4 and δ is by far not as impressive in the monkey as it is in the rat, indicating primarily phasic and not tonic inhibition.

In conclusion, the regional distribution of GABA_A_ receptor subunits is highly diverse in the monkey brain and less distinct than in rodents. Our findings thus indicate the expression of a high number of differently assembled receptors throughout the brain. The presumably different biochemical and physiological properties of individual receptor subtypes indicate a high level of differential fine‐tuning by these receptors. In the future, specific drugs may be developed for individually targeting receptor subtypes to interfere with their action at different sites of the brain.

## Data Availability

The data that support the findings of this study are available from the corresponding author upon reasonable request.
